# Helping hand for melanoma invasion: Transparent zebrafish can catch macrophages in the act

**DOI:** 10.1111/pcmr.12693

**Published:** 2018-03-06

**Authors:** Nikolay Ogryzko, Yi Feng

Metastatic melanoma ranks amongst the deadliest types of cancer, and metastasis itself is the primary killer in most solid tumours, yet gaps still exist in our understanding of this process. We know that innate immune cells, particularly tumour‐associated macrophage, play a significant role in promoting metastasis (Kitamura, Qian, & Pollard, 2015). Macrophage recruited to the tumour microenvironment is subject to a range of locally generated signals, which promote their switch to a pro‐tumourigenic phenotype. Many reciprocal signals mediate this interaction, including chemokines, cytokines, small metabolites and miRNAs (Noy & Pollard, 2014), but a thorough understanding of cell–cell interaction during this process has been hindered by the lack of an amenable model that allows the investigation of this process in vivo in real time.

The choice of zebrafish larval hindbrain ventricle is key to providing a reliable observation of metastasis in vivo in this study

In this study, Roh‐Johnson et al. establish a zebrafish larval xenograft melanoma model and image the interaction of macrophage with melanoma cells in vivo. The authors employ several new techniques to describe the unexpected mechanism of contact‐dependent cytoplasmic transfer which macrophage uses to promote melanoma cell invasion (Figure 1).

**Figure 1:**
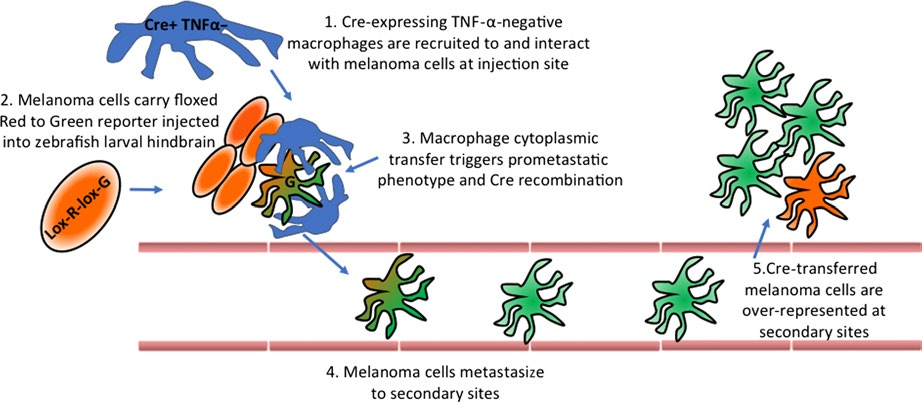
Macrophage cytoplasmic transfer correlates with melanoma cell metastasis [Colour figure can be viewed at wileyonlinelibrary.com]

The choice of zebrafish larval hindbrain ventricle is key to providing a reliable observation of metastasis in vivo in this study as it allows transplantation into a well‐isolated tissue, which is well served by the smaller circulatory vessels. Similar to the mammalian situation, cancer cells from here need to invade surrounding tissue, undergo intravasation and extravasation so as to seed in a distant site, such as the caudal hematopoietic tissue of the zebrafish. Furthermore, the transparent zebrafish larvae facilitate the visualization of cell–cell interaction during cancer cell metastasis, allowing the authors to describe the role of components of the immune system in establishing secondary tumours. Mirroring data from mammalian models, host macrophage recruitment to the primary xenograft of human melanoma cells enables tumour metastasis but is prevented by the removal of innate immune cells in pu.1‐negative larvae. Live imaging with a novel TNF‐α reporter also enabled the authors to classify the tumour‐associated macrophage spending a prolonged amount of time in contact with melanoma cells as having an alternatively activated phenotype as opposed to classically activated, proinflammatory macrophage.

From observing the cell‐to‐cell contact of macrophage and tumours, and supported by previous studies describing the potential of macrophage to transmit cytoplasmic material to other cell types (Saha et al., 2016), the authors hypothesized that this could be a mechanism responsible for a tumour‐associated macrophage promotion of metastasis. Using a macrophage‐delivered Cre recombinase (mpeg:cre) in combination with a xenograft of melanoma cells expressing a floxed red to green reporter, Roh‐Johnson et al. not only showed that some melanoma cells at the implantation site have been modified by macrophage cre, but more importantly, that a higher proportion of cells in secondary sites are recipients of cytoplasmic transferred cre (green) than cells not subject to the transfer (red). This implies that cancer cells contacted by macrophage and receiving macrophage cytoplasmic material are more likely to establish secondary tumours.

Further evidence for the process is provided by genetic modification of macrophage recruitment to the implantation site through the modification of the Toll‐like receptor adaptor molecule Myd88. Myd88‐negative zebrafish showed decreased macrophage recruitment to the implantation site, whereas macrophage expressing a constitutively active Myd88 showed greater macrophage infiltration. Downstream this resulted in a corresponding decrease or increase in tumour dissemination, respectively, demonstrating the role of macrophage in the process. With intravital time‐lapse imaging studies, a specific strength of the zebrafish model, the authors describe the correlation between macrophage and melanoma cell contact and subsequent melanoma metastasis, and are able to confirm these observations with an in vitro equivalent of the same model, also showing an increase in cancer cell directional persistence post‐cytoplasmic transfer. Finally, the macrophage cytoplasmic transfer to melanoma cells correlating with their metastatic potential is also confirmed in a murine lung metastasis model using subcutaneous injection of mouse melanoma B16F10 cells and genetic labelling of macrophage cytoplasmic transfer. The study provides a novel insight into the mechanisms by which the innate immune system, specifically macrophage, interact with the tumour to encourage metastasis, a mechanism potentially amenable to therapeutic intervention in treating cancer. Transfer of cytoplasmic material from macrophage is itself not a novel observation, but in the cancer context of macrophage aiding metastasis is an exciting discovery. The next question is the identity of pro‐metastatic factors involved. Conventional candidate molecules include matrix metalloproteinases, miRNAs and molecules regulating cell motility; however, transcription factors such as ZEB1 (Cortés et al., 2017), an inducer of epithelial to mesenchymal transition, are also strong candidates due to their potential to induce a pro‐metastasis transcription programme. Finding the causal factors would strengthen the conclusions of the article by providing a knockout target to demonstrate the causal link between macrophage cytoplasmic transfer and a more metastatic cell phenotype, an observation with a strong correlative rather than causative relationship. Moreover, the role of extracellular vesicles in pro‐metastatic mechanisms (Saha et al., 2016; Zomer et al., 2015) alongside this novel pathway and the varying contributions of each would give more insight into the biology of the metastatic process.

In this study, the authors use a highly innovative approach, with robust confirmation in murine and human models, to demonstrate a strong link between the pro‐growth functions of the innate immune system and cancer metastasis, providing strong parallels between regeneration and the promotion of tumour growth. This study greatly advances the field and provides exciting new avenues to address the cancer problem.

## CONFLICT OF INTERESTS

The authors declare no conflict of interests.

## References

[pcmr12693-bib-0001] Cortés, M. , Sanchez‐Moral, L. , de Barrios, O. , Fernández‐Aceñero, M. J. , Martínez‐Campanario, M. , Esteve‐Codina, A. , … Postigo, A. (2017). Tumor‐associated macrophages (TAMs) depend on ZEB1 for their cancer‐promoting roles. The EMBO Journal, 36(22), 3336–3355. 10.15252/embj.201797345 29038174 PMC5686549

[pcmr12693-bib-0002] Kitamura, T. , Qian, B. Z. , & Pollard, J. W. (2015). Immune cell promotion of metastasis. Nature Reviews Immunology, 15(2), 73–86. 10.1038/nri3789 PMC4470277 25614318

[pcmr12693-bib-0003] Noy, R. , & Pollard, J. W. (2014). Tumor‐associated macrophages: From mechanisms to therapy. Immunity, 41(1), 49–61. 10.1016/j.immuni.2014.06.010 25035953 PMC4137410

[pcmr12693-bib-0004] Saha, S. , Aranda, E. , Hayakawa, Y. , Bhanja, P. , Atay, S. , Brodin, N. P. , … Pollard, J. W. (2016). Macrophage‐derived extracellular vesicle‐packaged WNTs rescue intestinal stem cells and enhance survival after radiation injury. Nature Communications, 7, 1–16. 10.1038/ncomms13096 PMC5065628 27734833

[pcmr12693-bib-0005] Zomer, A. , Maynard, C. , Verweij, F. J. , Kamermans, A. , Schäfer, R. , Beerling, E. , … Van Rheenen, J. (2015). In vivo imaging reveals extracellular vesicle‐mediated phenocopying of metastatic behavior. Cell, 161(5), 1046–1057. 10.1016/j.cell.2015.04.042 26000481 PMC4448148

